# Bacillus Calmette-Guerin 's beneficial impact on glucose metabolism: evidence for broad based applications

**DOI:** 10.1016/j.isci.2021.103150

**Published:** 2021-09-21

**Authors:** Gabriella F. Shpilsky, Hiroyuki Takahashi, Anna Aristarkhova, Michele Weil, Nathan Ng, Kacie J. Nelson, Amanda Lee, Hui Zheng, Willem M. Kühtreiber, Denise L. Faustman

**Affiliations:** 1 Massachusetts General Hospital, Immunobiology Laboratories, Boston, MA 02129, USA; 2 Massachusetts General Hospital and Harvard Medical School, Immunobiology Laboratories, Boston, MA 02129, USA; 3 Massachusetts General Hospital, Diabetes Unit, Boston, MA 02129, USA; 4 Massachusetts General Hospital and Harvard Medical School, Statistics Department, Boston, MA 02129, USA

**Keywords:** Human metabolism, Immunology

## Abstract

Bacillus Calmette-Guerin (BCG) vaccinations improve glycemic control in juvenile-onset Type I diabetes (T1D), an effect driven by restored sugar transport through aerobic glycolysis. In a pilot clinical trial, T1D, but not latent autoimmune diabetes of adults (LADA), exhibited lower blood sugars after multidose BCG. Using a glucose transport assay, monocytes from T1D subjects showed a large stimulation index with BCG exposures; LADA subjects showed minimal BCG-induced sugar responsiveness. Monocytes from T1D, type 2 diabetes (T2D), and non-diabetic controls (NDC) were all responsive in vitro to BCG by augmented sugar utilization. Adults with prior neonatal BCG vaccination show accelerated glucose transport decades later. Finally, in vivo experiments with the NOD mouse (a T1D model) and obese db/db mice (a T2D model) confirm BCG's blood-sugar-lowering and accelerated glucose metabolism with sufficient dosing. Our results suggest that BCG's benefits for glucose metabolism may be broadly applicable to T1D and T2D, but less to LADA.

## Highlights

• A pilot trial of BCG vaccinations to T1D showed reduced blood sugars but not in LADA
• Monocytes from T1D and to some degree T2D display stimulated glucose transport
• BCG vaccinations at birth show accelerated glucose transport decades later
• In vivo mouse models of both T1D and T2D demonstrate BCG-induced blood sugar lowering

## Graphical abstract

**Figure:**
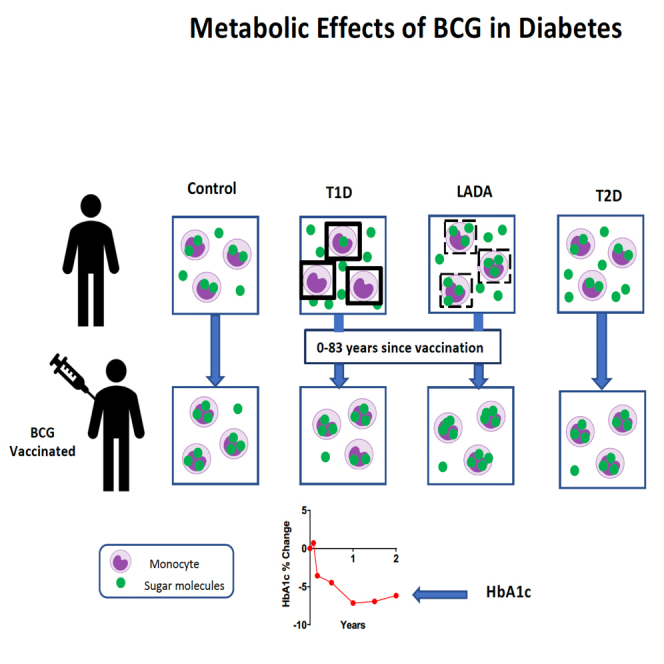

## Introduction

The BCG (Bacillus Calmette-Guerin) vaccine has been in clinical use for >100 years for protection from tuberculosis (Colditz et al., 1994). Over the last 15 years, diverse clinical trials have established that this microbe-based vaccine has additional clinical benefits called heterologous effects, off-target effects, or nonspecific effects. These terms indicate beneficial human clinical effects for diseases other than tuberculosis (de Bree et al., 2018; Freyne et al., 2015; Goodridge et al., 2016; Kandasamy et al., 2016; Pollard et al., 2017; Shann, 2010; Yung, 2016). For instance, the BCG vaccine protects humans from being infected with or dying from a range of infections, including upper respiratory tract infections, malaria, yellow fever, among others (Aaby et al., 2006, 2011; de Castro et al., 2015; Higgins et al., 2016; Hollm-Delgado et al., 2014; Kjaergaard et al., 2016; Setia et al., 2006; Stanley et al., 1981; Stensballe et al., 2005). The BCG vaccine also protects, halts, or reverses established autoimmunity. BCG used in early-stage multiple sclerosis shows, after a 2-year interval, a halt in disease progression in double-blinded clinical trials (Ristori et al., 1999, 2014). Repeat BCG vaccinations in longstanding T1D lower blood sugars in clinical trials (Faustman et al., 2012; Kühtreiber et al., 2018). Lastly, the BCG vaccine has been used globally for the past 40 years to treat bladder cancer and, more recently, to treat lymphoma and lung cancer (Babjuk et al., 2017; Davignon et al., 1971; Morales et al., 1976; Odom et al., 1988; Salmon et al., 2019, 2020; Usher et al., 2019).

The mechanisms behind these beneficial heterologous effects are under investigation. Abundant evidence has established that BCG works through the innate immune system on monocytes by rapidly changing their cytokine expression. The effect may be controlled by increased histone 3 lysine 4 tri-methylation (Arts et al., 2018; Biering-Sorensen et al., 2018). These changes are referred to as trained immunity, a type of host-microbe interaction known to occur in plants, invertebrates, and mice. Characteristically, a change in histone methylation has been established to rapidly change cytokine expression patterns. This may be a mechanism for BCG's rather rapid protection from infectious diseases (Aaby et al., 2006, 2011; Biering-Sorensen et al., 2018; de Castro et al., 2015; Higgins et al., 2016; Hollm-Delgado et al., 2014; Kjaergaard et al., 2016; Arts et al., 2018; Setia et al., 2006; Stanley et al., 1981; Stensballe et al., 2005).

The mechanisms behind BCG's beneficial systemic effects in humans also involve the adaptive immune response, and these changes appear to occur at a slower rate over years. This process of adaptive immune training can involve T cells as well as monocytes and centers on metabolic pathways that change aerobic glycolysis. Thus, the use and rate of sugar metabolism is improved after BCG therapy (Kühtreiber et al., 2018, 2020). The BCG effects observed in human autoimmune clinical trials have a slower onset of disease modifications (over years) but are permanent for >8 years in treated humans (Kühtreiber et al., 2018). Clinical examples of adaptive training include the reprogramming of T-regulatory (or Treg) cells by demethylation of key genes involved in their cellular activation (Kühtreiber et al., 2018). Another clinical example of adaptive training is the systemic reprogramming of T cells and monocytes in metabolic pathways related to aerobic glycolysis, a pathway with T1D-inherent defects (Kühtreiber and Faustman, 2019). The durability and the long-lasting effects of BCG vaccination have been also attributed to Th1/Th17 responses (Kleinnijenhuis et al., 2014).

Diabetes, a clinical form of high blood sugars, comes in several forms. Type 1 diabetes (T1D) is also commonly known as juvenile-onset diabetes (Katsarou et al., 2017). Its onset is typically in childhood, with rapid loss of pancreas function from the T cell autoimmune attack on the insulin-secreting islets of Langerhans. T1D requires life-long insulin therapy and is associated with serious morbidity and mortality. The children produce autoantibodies to various islet-related proteins. Diabetes can also occur in adulthood in two different forms. Late autoimmune disease of adults (LADA), also called Type 1.5 diabetes, is associated with an older age of onset (AOO), always greater than 21 years, with a mean age of onset of 30 years (Pieralice and Pozzilli, 2018). LADA subjects have a slower decline in pancreas function and also become insulin dependent. The most prevalent form of diabetes is Type 2 diabetes (T2D). This form of diabetes typically occurs in adults with a mean onset age of 30–50 years and is associated with obesity and insulin resistance. With time, T2D is treated with insulin but more commonly is treated with oral hypoglycemia agents, such as metformin. The genetics of T2D are distinct from the genetics of T1D. The “glue” linking all three forms of diabetes is high blood sugar.

This study explores the breadth of BCG-induced sugar-lowering and accelerated glucose transport properties across all forms of diabetes and nondiabetic controls (NDCs). We start with a two-year pilot clinical trial comparing T1D with LADA subjects for blood sugar control after multidoses of the BCG vaccine. Then, using an in vitro assay of glucose metabolism to monitor the rate of cellular glucose uptake (2-NBDG), we examine BCG's effect on sugar uptake by monocytes from all forms of diabetes plus NDCs. We then examine the durability of this BCG effect by studying adults who received the BCG vaccine as neonates. Lastly, we turn to murine models of T1D and T2D to determine whether BCG's beneficial effect on sugar metabolism is equally observed in two forms of divergent hyperglycemia. To confirm that BCG-induced accelerated glucose transport is due to glycolysis, the inhibitor metformin is used in vitro and in vivo (Arts et al., 2016; Kühtreiber et al., 2020).

## Results

**Figure 1:**
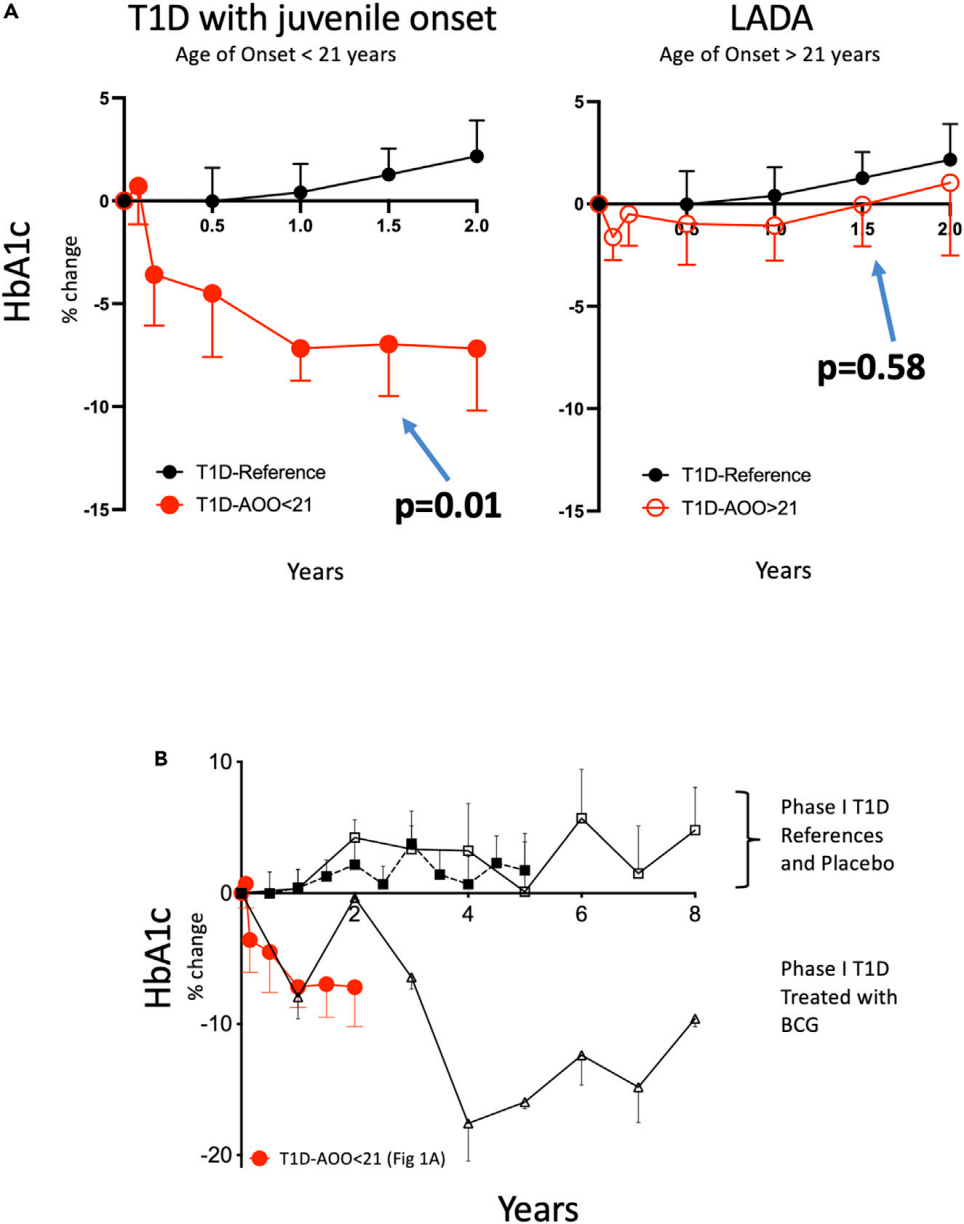
In vivo BCG vaccinations decrease HbA1c in T1D but not LADA patients (A) Juvenile-onset T1D diabetic subjects were compared with LADA diabetic subjects for responsiveness to BCG vaccines using the Tokyo strain over a 2-year period. Juvenile-onset diabetes was defined as age of onset <21 years. LADA subjects were defined as having diabetes onset >21 years. Percent change from baseline in HbA1c of open-label T1D patients receiving BCG treatment with an age of onset (AOO) ≤ 21 years old (n = 6) (mean age of onset of 11 ± 3 years) compared with a T1D reference population not receiving BCG treatment. The fall in HbA1c is a significant trend (repeated-measures ANOVA p = 0.01; left). The trend in the LADA patients was not significant (n = 10; repeated-measures ANOVA p = 0.58; right). Their mean age of onset was 31 ± 2 years. The current chronological ages of the two adults groups were for 28 ± 3 years for the T1D and 45 ± 4 years for LADA subjects. The duration of diabetes in the T1D group was 18 ± 3 years, and the duration of diabetes in the LADA group was 19 ± 2 years. (B) Percent change from baseline in HbA1c of the current open-label T1D patients with AOO ≤21 years (n = 6; 11 ± 3.0 years) receiving Tokyo BCG treatment is shown in red (n = 6) as compared with the previously published Phase 1 Sanofi BCG clinical trial data with similarly early onset (11 ± 5.8 years) (black, open triangle). Also shown are the Phase 1 placebo group (open squares, and a reference population (closed squares).

**Figure 2:**
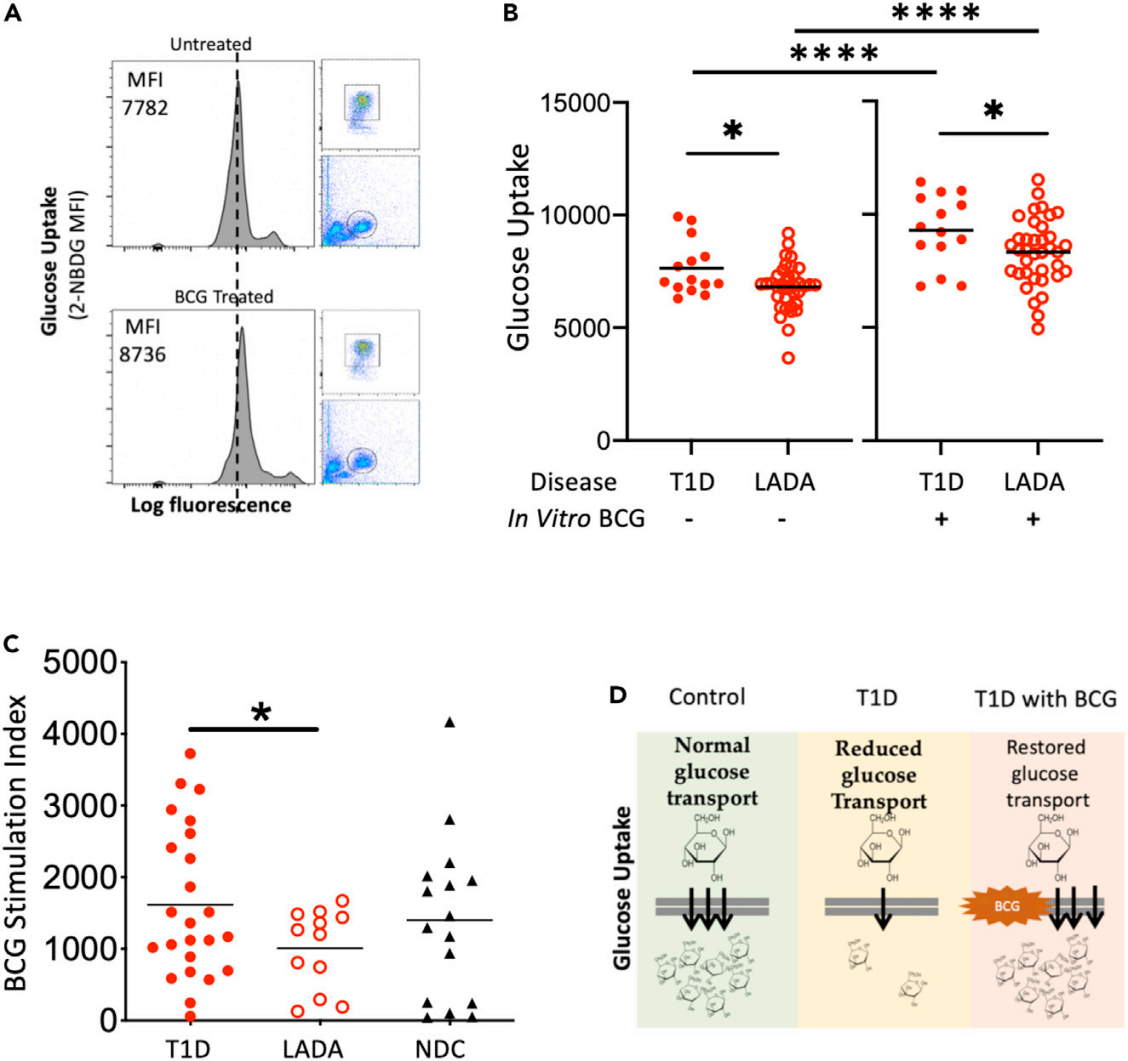
Glucose transport by T1D, LADA, and NDC human monocytes (A) Example of flow cytometry showing gating and glucose uptake measured with 2-NBDG MFI analysis of nondiabetic control (NDC) monocytes treated in vitro with BCG for 24 hours and then allowed to transport 2-NBDG (sugar) for 1 hour. (B) LADA (red open dots) and T1D (red closed dots) monocytes differ both at baseline (left) and after the in vitro BCG exposure (right) with respect to glucose transport. At baseline, T1D monocytes' basal glucose transport was 7,641 ± 325 and LADA monocytes' was 6,804 ± 170. After BCG, the MFI was 9,299 ± 421 for T1D and 8,331 ± 240 for LADA (p < 0.05 for both comparisons). At baseline LADA have insufficient glucose transport compared with augmented baseline transport in T1D. The comparison of T1D +/− BCG and of LADA +/− BCG is both significant at p < 0.0001. The number of subjects: T1D n = 14; LADA n = 37. (C) We next measured the stimulated glucose index of monocytes from subjects treated with BCG in vivo. As presented above, T1D subjects had improved blood sugar control with BCG but LADA subjects at year 2 had no improvement in their blood sugar control measured in the HbA1c assay. Comparison of the BCG stimulation index (stimulated glucose uptake - baseline) from isolated monocytes from the clinical trial subjects shows increased glucose uptake and greater accelerated uptake of glucose in T1D as compared with LADA. There was no significant difference between the BCG stimulation index of T1D and NDC. Red closed dots represent T1D monocyte samples; black triangles represent nondiabetic control monocytes, open red dots represent LADA monocyte samples. Student's t-testing (unpaired, 1-tailed) or a student's t-testing (paired, 1-tailed) was represented as: p < 0.05 ∗; p < 0.01 ∗∗; p < 0.001 ∗∗∗, p < 0.0001∗∗∗∗. An unpaired t test was used comparing T1D with either LADA or controls. A paired test was used comparing internal to self as untreated monocytes to BCG treated monocytes. The number of subjects: T1D n = 24; LADA n = 12; NDC n = 16. (D) Summary depiction of how, after in vitro BCG exposure, the 2-NBDG sugar uptake assay detects measurable changes in monocytes' sugar transport.

**Figure 3:**
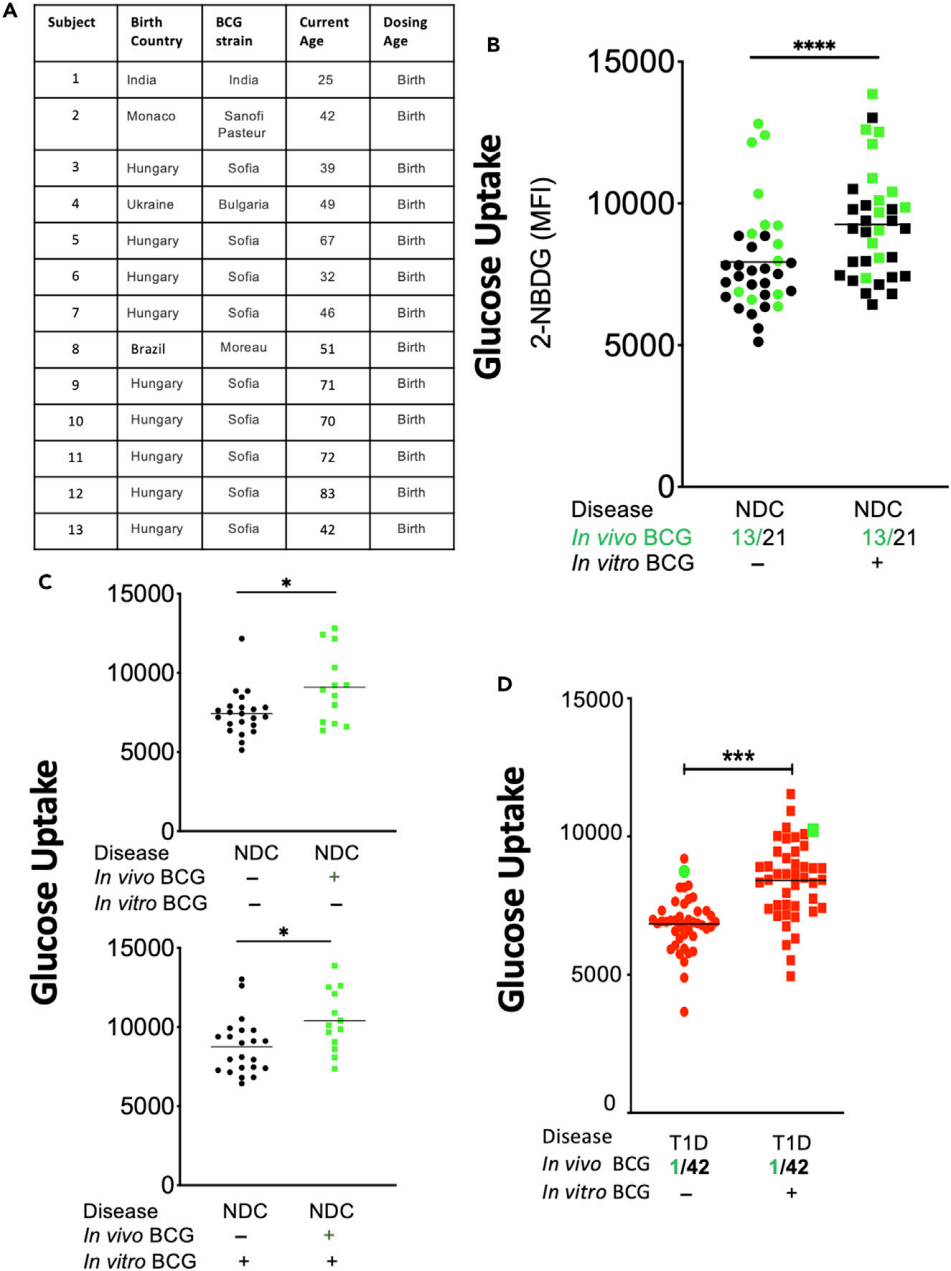
Neonatal BCG vaccinations show sustained and lifelong changes in glucose metabolism (A) Demographics of control subjects vaccinated with BCG at birth is presented by the country of birth, the BCG strain used in that county, and the current age of subjects (a reflection of years since the vaccination). (B) Glucose uptake in monocytes from nonvaccinated control subjects (n = 21, black dots) compared with neonatal BCG vaccinated control subjects (n = 13, green dots). Data present baseline uptake of sugar by monocytes (left) compared with sugar uptake after 24 hour in vitro exposure to BCG (right). (C) Baseline (top) and BCG-stimulated (bottom) monocyte glucose uptake of NDC patients (n = 21) compared on the same scale as NDC subjects given neonatal BCG vaccinations (n = 13) (2-tailed, unpaired t test revealed a significant difference at baseline and with stimulation. Both baseline sugar uptake (top, green) and BCG-stimulated sugar uptake (bottom, green) were monocyte characteristics from normal controls vaccinated at birth with BCG. (D) Baseline (left) glucose uptake in monocytes of unvaccinated T1D patients (n = 42) following a one day incubation with or without in vitro BCG treatment (right). The sample marked in green represents a T1D individual with remarkable diabetes management and a mass found in their lungs suspicious for latent *Mycobacterium tuberculosis* infection. p < 0.05 ∗; p < 0.01 ∗∗; p < 0.001 ∗∗∗

**Figure 4:**
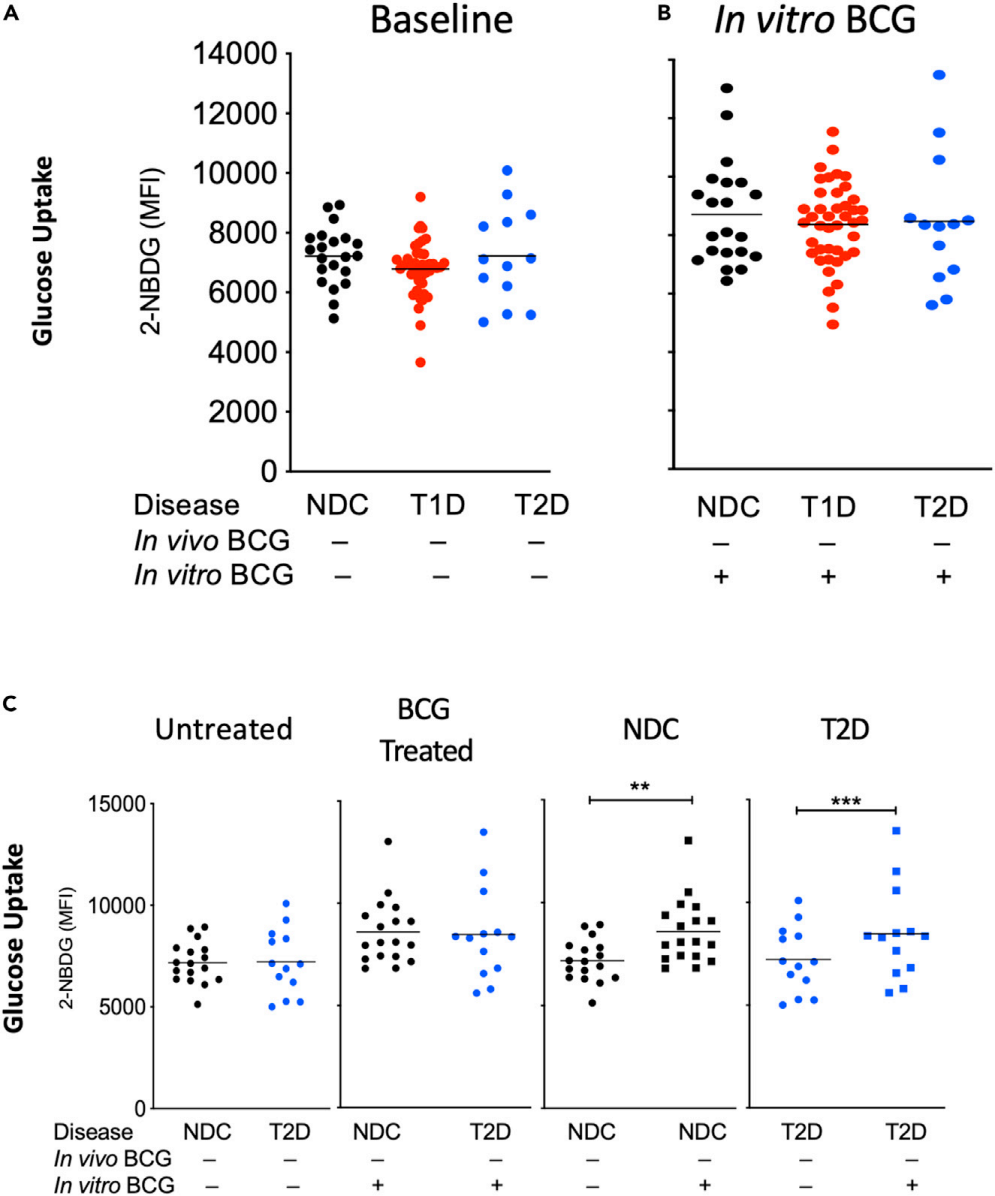
T2D monocytes transport more sugar with exposures to BCG in culture, in many ways their glucose uptake profile is similar to NDC (A) Untreated monocytes at baseline showing glucose uptake for NDC (n = 21), T1D (n = 42), and T2D (n = 13) subjects. (B) Glucose uptake for monocytes from NDC, T1D, and T2D patients after in vitro treatment with BCG for 24 hrs. (C) Comparison of glucose uptake by monocytes from NDC (n = 17) and T2D (n = 13). Overnight culture in BCG increased glucose uptake in both NDC and T2D monocytes. p < 0.05 ∗; p < 0.01 ∗∗; p < 0.001 ∗∗∗. Red dots represent T1D monocyte samples; black dots represent nondiabetic control (NDC) monocytes, and blue dots represent T2D monocyte samples.

**Figure 5:**
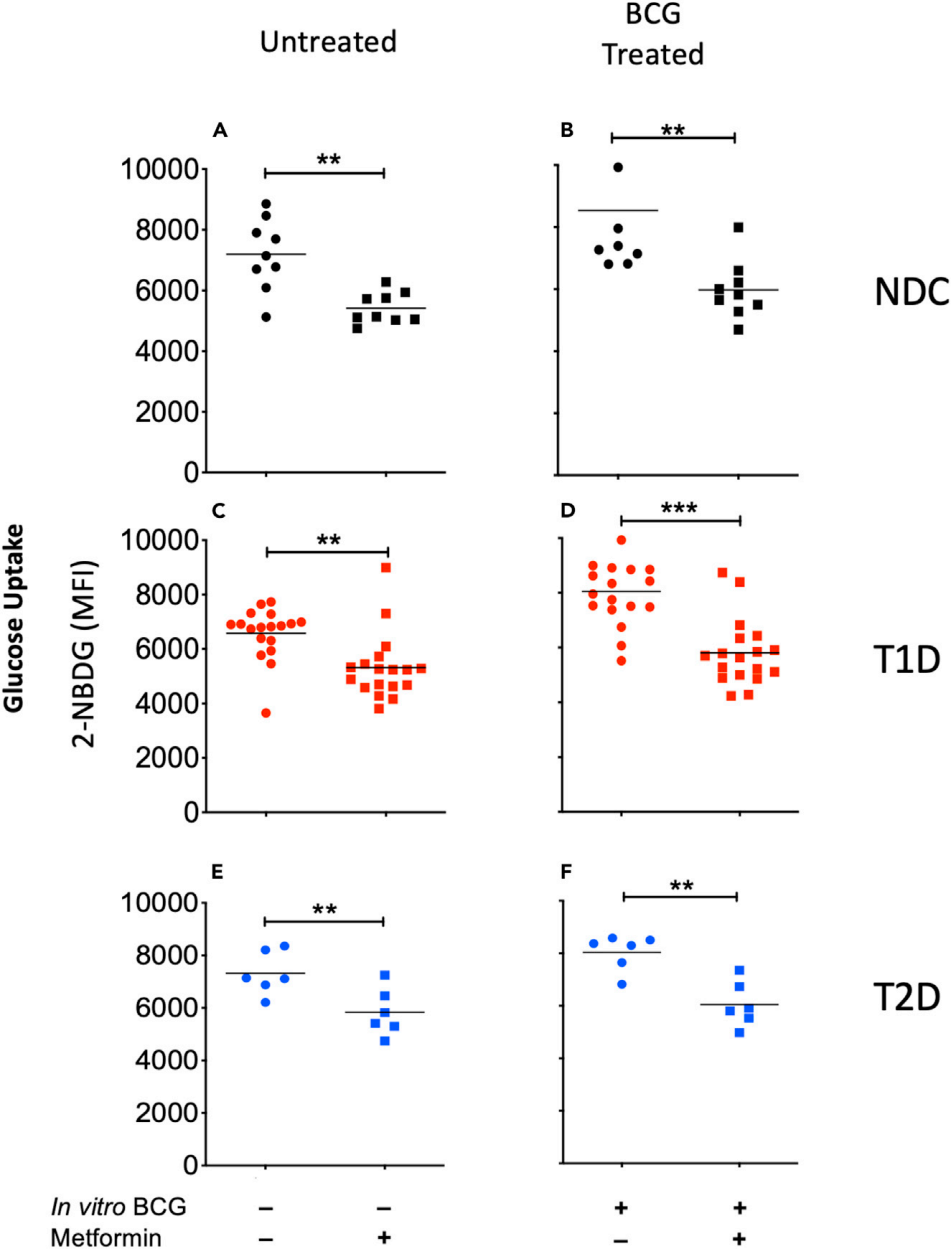
Metformin inhibits glucose uptake in cultured NDC, T1D, and T2D human primary monocytes (A) Metformin inhibited glucose uptake (2-NBDG) in untreated NDC monocytes (n = 9, p = 0.00075). (B) Metformin inhibited glucose uptake (2-NBDG) in NDC monocytes cultured in the presence of BCG (n = 9, p = 0.0047). (C) Metformin inhibited glucose uptake (2-NBDG) in untreated T1D monocytes (n = 18, p = 0.0014). (D) Metformin inhibited glucose uptake (2-NBDG) in T1D monocytes cultured in the presence of BCG. n = 18, p = 3.11 × 10^−6^). (E) Metformin inhibited glucose uptake (2-NBDG) in untreated T2D monocytes (n = 6, p = 0.014). (F) Metformin inhibited glucose uptake (2-NBDG) in T2D monocytes cultured in the presence of BCG (n = 6, p = 0.0012). All p values are from paired, 2-tailed student's t test. p < 0.05 ∗; p < 0.01 ∗∗; p < 0.001 ∗∗∗. Red dots represent T1D monocyte samples, black dots represent nondiabetic control monocytes, and blue dots represent T2D monocyte samples.

**Figure 6:**
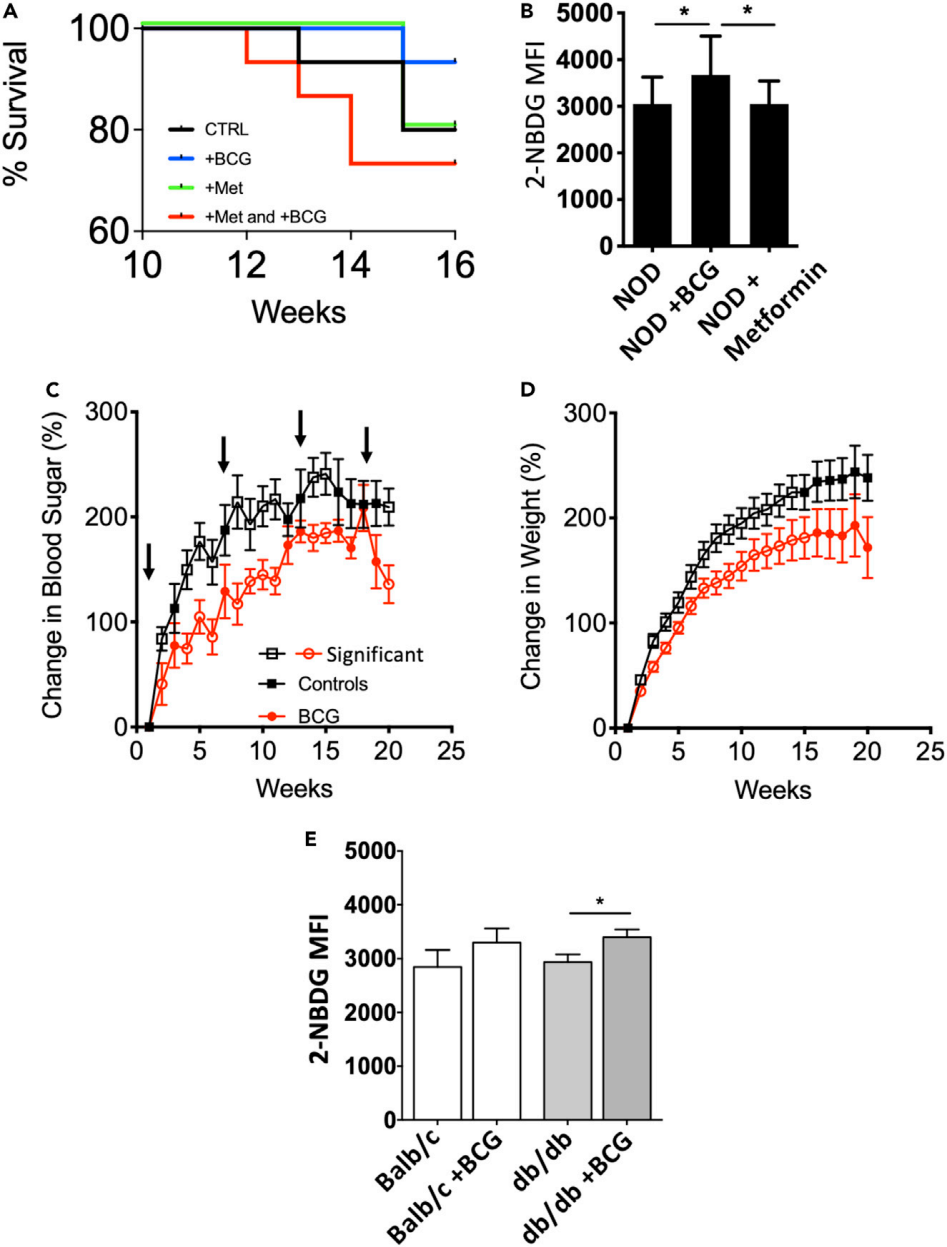
Mouse models of T1D and T2D support BCG therapeutic effects; in vitro glucose transport assays are consistent (A) Kaplan Meyer survival plot for NOD mice that were untreated (CTRL n = 16), treated with BCG only (+BCG n = 11), treated with metformin only (+Met n = 15), or treated with metformin and BCG (+Met and +BCG n = 15). Metformin treatment started at 6 weeks of age in vivo; BCG injections were administered at week 7 and week 13. Survival was improved after treatment with BCG, but treatment with metformin alone or with a combination of metformin plus BCG resulted in reduced animal survival due to severe hyperglycemia. (B) Glucose uptake (2-NBDG) studies in isolated bone marrow cells from untreated, BCG-treated, or metformin-treated diabetic NOD mice show significant differences between untreated and BCG-treated mice (p = 0.015), as well as between BCG-treated and metformin treated mice (p = 0.011). In all cases, metformin was started at 6 weeks of age, and this was then followed by BCG treatment at 10 weeks. The number of mice: NOD n = 16; NOD + BCG n = 11; NOD + metformin n = 15. (C) Change in blood sugar and BCG treatment regimen for obese db/db mice, a model of T2D. Arrows indicate BCG treatment times. The BCG-treated mice have substantially lower blood sugars. Open symbols depict time points where the difference between CTRL and BCG groups was significant in Student's t testing. Number of mice: db/db + BCG n = 16; db/db untreated n = 11. (D) Change in body weight in the severely obese db/db mice suggests that BCG-treated mice are healthier since they have lower body weight. Open symbols depict time points where the difference between CTRL and BCG groups was significant in student's t testing. The number of mice: db/db + BCG n = 16; db/db untreated n = 11. (E) In vivo BCG treatment of normal BALB/c mice and of diabetic db/db mice both result in increased glucose uptake although the Balb/c mouse effect did not reach statistical significance as measured by 2-NBDG assays in bone marrow cells compared to untreated control mice. BALB/c: n = 4 + BCG and n = 4 untreated; p = 0.31; db/db: n = 10 BCG and n = 10 untreated; p = 0.04.

This is a study of metabolic changes induced by the BCG vaccine in diabetic humans. This study, both in vivo and in vitro, quantifies sugar metabolism (n = 391 subjects). In a pilot clinical trial of 62 adults followed up for 2 years, the HbA1c response in T1D subjects (age of onset ≤21), LADA subjects (age of onset >21), and untreated controls were evaluated (Figure 1). In in vitro studies, the 2-NBDG sugar utilization assay data quantified the systemic effects of BCG. The magnitude of sugar metabolic changes was in cells from in vivo BCG treatments, from in vitro BCG exposures, and in the setting of T1D, LADA, T2D, neonatal BCG vaccinations, and bladder cancer subjects (total n = 329 subjects) (Figures 2, 3, 4, 5, and S1). Diabetic mouse studies were performed to confirm similar mechanisms of BCG-induced alterations in metabolism that lower blood sugars (n = 112 mice; Figure 6).

### Patients with T1D, but not LADA, respond to BCG vaccinations by HbA1c lowering within 2 years

T1D is commonly referred to as juvenile-onset diabetes based on childhood onset. Herein we define T1D as having an AOO ≤ 21 years. Adults can also get T1D with older AOO, herein defined as AOO >21 years, and marked by slower progression to total insulin dependence, some characteristics of T2D often with insulin resistance, obesity, and slower C-peptide decline. This latter form of diabetes is called LADA. Published data in a small, double-blinded placebo-controlled Phase I clinical trial showed juvenile-onset T1D was responsive to repeat BCG vaccinations with lowered HbA1c values. These adults received the Sanofi BCG strain (Kühtreiber et al., 2018).

We performed an open-label pilot clinical trial to investigate the reproducibility of these early observations in T1D, to test if Tokyo BCG strain shows a similar HbA1c lowering trend as the earlier Sanofi BCG studies, and to see if LADA subjects are equally responsive to the BCG intervention over the same time course as T1D. T1D (AOO ≤21; n = 6; repeated-measures analysis of variance [ANOVA] p = 0.01) (Figure 1A, left), when treated in adulthood, showed uniform reductions in HbA1c over the two-year period as compared with baseline (Figure 1A, left). In contrast, LADA subjects over this two-year observation period did not reduce their HbA1C values (n = 10; repeated measure ANOVA p = 0.58) (Figure 1A, right). An untreated T1D reference population (n = 40) is also provided for context. Note that the data shown were normalized by calculating % change in HbA1c from baseline. The raw data are shown in Figure S2. Our findings suggest that T1D diabetic subjects (vs LADA) are most sensitive to BCG vaccination, are more homogeneous in their response, have a greater magnitude response, and/or are at least are faster in their blood sugar lowering responses with observations out to two years.

Because there are known differences in BCG strains, the current data from the Tokyo BCG strain were compared with the Sanofi-strain-treated Phase I double-blinded clinical trial data, a data set having similar early AOOs. We compared the change in HbA1c from the current open-label juvenile-onset T1D (n = 6) cohort with our Phase 1 trial composed of juvenile-onset BCG-treated T1D cohort, the original reference and original placebo groups T1D Population with the same number of BCG doses (Figure 1B). The data show similar kinetics and uniformity of all adults with juvenile-onset T1D dosed with either the Sanofi or the Tokyo BCG strain. Both strains show a drop in HbA1c levels over 2 years, indicating reproducibility.

### Monocytes from T1D and NDCs but not LADA show accelerated sugar uptake to varying degrees

We next tested the ability of a glucose transport assay, 2-NBDG (fluorescent glucose analogue, C_12_H_14_N_4_O_8_), to corroborate the clinical trial observations. The 2-NBDG sugar uptake assay is a monitoring tool that measures enhanced intracellular monocyte fluorescence when the labeled sugars are transported inside the cell (Figure 2A). Monocytes were isolated from peripheral blood and cultured overnight with or without BCG. The cells were then harvested, washed, counted, incubated with 2-NBDG for 1 hour at 37°C, and labeled with APC-anti-CD14 antibody. The cells were then analyzed using a BD FACSCanto II flow cytometer. The right shift in the histogram for 2-NBDG fluorescence on this log scale indicates that the uptake of 2-NBDG (labelled sugar) into the BCG-treated cells is higher than that into untreated control cells. The outcome is quantified by median fluorescent intensity (MFI). A demographics table for the patients who donated blood for these experiments is shown (Table S1).

At baseline (i.e., freshly isolated monocytes cultured overnight in the *absence* of BCG), sugar uptake measured by the 2-NBDG assay showed that monocytes from T1D subjects had greater sugar transport than monocytes from LADA subjects (T1D n = 14 and LADA n = 37; Figure 2B, left). When cultured overnight in the presence of BCG, both T1D and LADA monocytes accelerated their sugar transport, but T1D monocytes were much more responsive than LADA monocytes (p = 0.03).

We next studied freshly isolated monocytes from clinical trial subjects, either T1D or LADA, for in vitro sugar transport *after* BCG treatment in vivo. These assays were performed two years into the human clinical trial. The BCG stimulation index (BCG treatment in vitro minus untreated monocytes in vitro) rapidly demonstrated that a large augmented sugar transport was specific for T1D compared with LADA (n = 24, 12, and 16 for T1D, LADA, and NDC, respectively; Figure 2C). Thus, 2-NBDG uptake correlated with the clinical responsiveness of BCG administered in vivo in T1D but not LADA subjects.

A summary of the 2-NBDG assay and the glucose uptake assay for measuring the labelled sugar is presented in Figure 2D.

### Glucose uptake is accelerated decades after neonatal BCG vaccinations

Can accelerated sugar metabolism be observed decades after neonatal BCG vaccination? The subjects used for this next study were adults vaccinated at birth with BCG (Figure 3A). Their newborn vaccinations were performed in diverse countries and with various strains of BCG. The time intervals from vaccination to our in vitro glucose uptake assay ranged from 25–83 years. The data show the impact of prior BCG vaccinations at birth on uptake of glucose compared with nonvaccinated adult subjects (NDC, n = 21 subjects; neonatal vaccinations n = 13). The neonatal vaccinated nondiabetic subjects on average had BCG vaccination 55.3 ± 4.5 years before this blood monocyte study. Remarkably, the monocytes from neonatal BCG-vaccinated normal adult subjects (green dots) continued to have accelerated sugar transport both at baseline and after in vitro BCG compared with NDC samples (Figure 3B, left, green dots compared with black dots). The trends were statistically significant at baseline and with BCG stimulation compared with nonvaccinated control monocytes (Figure 3B; NDC with childhood BCG vaccination n = 13 versus unvaccinated n = 21). With neonatal vaccinations in controls, the baseline monocyte glucose uptake was 9,099 ± 626; the nonvaccinated control baseline glucose uptake was 7,433 ± 303 (Figure 3C, top). With monocyte culture with BCG for 24 hours, neonatal vaccinations in controls had stimulated glucose uptake in monocytes of 10,397 ± 537. The nonvaccinated control stimulated BCG glucose uptake was 8,746 ± 377 (Figure 3C, bottom). The findings suggest lifelong persistence of BCG's metabolic effects.

The other clinical setting in which BCG is administered is as high-dose therapy for bladder cancer, almost always in elderly populations. We asked, could normal subjects treated with high doses of BCG for bladder cancer also show changes in glucose metabolism, measured in vivo with HbA1c or through monocyte glucose uptake assays in the form of 2-NBDG. High-dose BCG is administered in the bladder. Subjects were studied months to 20 years after bladder cancer treated with BCG (Figure S1). Unlike neonatal vaccination, accelerated glucose transport by monocytes at baseline and after in vitro BCG exposures were not observed. All these subjects were US citizens and based on medical records received the TICE BCG strain as the bladder drug, a strain with less potency than other BCG strains. In addition, the stage of bladder cancer may also determine if the BCG from bladder erosions for the cancer will allow systemic population/spread of the administered BCG. These data were not feasible to obtain for this study.

We were fortunate to identify a T1D subject in our clinic with lung granulomas consistent with a latent form of mycobacteria infection for >30 years and T1D for 52 years. His clinical course was remarkable for having lifelong near-perfect HbA1c control, always in the 5.5 to 6.5 range. Sampling of his monocytes revealed augmented sugar transport, thus showing that even tuberculosis itself might be as protective as the BCG vaccine; however this is a single case (Figure 3D, green dots).

### T2D monocytes are responsive to BCG exposures in vitro

The third form of diabetes, T2D, was studied using the in vitro glucose uptake assay to determine responsiveness to BCG (Figure 4). The data show that T2D monocytes at baseline have adequate sugar transport and also could respond in vitro to BCG exposures (n = 21 NDC, n = 42 T1D, n = 13 T2D, respectively [Figures 4A and 4B]). Further comparisons between NDC and T2D monocytes are also presented (Figure 4C). There was no difference between NDC and T2D, either at baseline (untreated panel) or after culture with BCG (BCG-treated panel).

### Metformin inhibits BCG-induced glucose uptake

Metformin is taken by many T2D patients to help control their blood sugars by increasing insulin sensitivity and by inhibiting glucose neogenesis by the liver. Metformin is also taken by a small fraction of T1D. It is therefore important to establish whether metformin interferes with BCG with respect to glucose uptake in T1D, as well as T2D in preparation for a potential use of BCG in T2D clinical trials. We therefore studied the effect of metformin and BCG in vitro in human monocytes (Figure 5). Previous data suggest that metformin interferes with cytokines induced by BCG through innate immunity as well as with glycolysis pathways in innate and adaptive immunity (Arts et al., 2016; Kühtreiber et al., 2020).

Using the glucose metabolic assay, 2-NBDG, treatment with metformin in vitro inhibited 2-NBDG uptake in primary human monocytes from all the clinical samples (NDC, T1D, and T2D). We isolated primary monocytes from NDC, T1D, and T2D subjects and cultured them overnight in the presence or absence of BCG and metformin. The next day the cells were incubated with 2-NBDG and APC-anti-CD14 antibodies and analyzed by flow cytometry. For all forms of diabetes, metformin reduced 2-NBDG uptake by the monocytes, irrespective of whether BCG was present (Figure 5). The number of subjects: 5A, B n = 9; 5C, D n = 18; 5E, F n = 6. We thus conclude that in vitro metformin inhibits 2-NBDG uptake in both untreated and BCG treated monocytes and thus may interfere with the beneficial effects of BCG treatment in vivo for blood sugar control in diabetics. Metformin has its own benefits for blood glucose control in diabetes, its effect is not likely additive with the effect of BCG, and it may not be possible to combine both treatments.

### Murine T1D model (NOD mouse) responds to BCG; metformin inhibits sugar metabolism in vivo

We performed experiments in the NOD mouse, a well-established animal model of T1D. Prediabetic (4 weeks old) NOD mice were divided into four groups based on treatment with BCG injections and/or metformin (added to mouse chow) (n = 57 NOD mice). Specifically, the mice were treated with one dose of BCG only, metformin only, both BCG and metformin, or no treatment (see STAR Methods for details) (Figure 6). The results presented as Kaplan-Meier survival curves for the NOD mice show that BCG increases survival compared with the untreated control group of mice or NOD mice treated with only metformin. In contrast, the combination BCG and metformin accelerated death in these mice (Figure 6A). The BCG-only treated mice had the highest survival rate, followed by the untreated controls and the metformin-treated mice. The lowest survival rate was achieved in the group of NOD mice that were treated with both BCG and metformin, again suggesting that BCG and metformin treatments should not be combined. In the mouse, sufficient quantities of peripheral monocytes cannot be isolated, so instead we used freshly isolated bone marrow cells from the NOD mice, in which we studied sugar transport by the 2-NBDG assay (Figure 6B). As expected, BCG significantly increased the 2-NBDG uptake into freshly isolated bone marrow cells from NOD mice (n = 11) as compared with untreated NOD mice (n = 16, p = 0.015) and also as compared with metformin-treated NOD mice where metformin inhibited the BCG-induced accelerated glycolysis (n = 15, p = 0.011).

### Obese db/db mice, a model of T2D, respond to BCG with lowered sugars and reductions in obesity

We also performed BCG experiments in the BKS db/db mice, a model of T2D. The mice were divided into 2 groups. One group was treated with multiple BCG injections, whereas the other group was untreated. Changes in blood sugars and weight were monitored (Figures 6C and 6D). Blood sugars for the BCG-treated db/db (n = 16) were significantly lower than for the untreated db/db mice (n = 11). The corresponding weight reduction from their severe obesity is shown (Figure 6D). The open symbols depict time points where the difference between CTRL and BCG groups was significant in student's t testing. Because weight gain and hyperglycemia go hand-in-hand in db/db mice, the lower weight in the BCG-treated mice suggests better health and presumably better glycemic control. It should be noted that early db/db mouse experiments tried one single dose of BCG; that dose failed to have a clinical outcome. Therefore, subsequent studies delivered four BCG doses.

We isolated bone marrow cells to test lymphoid sugar transport from all BCG-treated and untreated db/db mice and performed 2-NBDG uptake experiments (Figure 6E). For both db/db and control mouse strains, the in vivo BCG treatments resulted in bone marrow cells that were capable of increased 2-NBDG uptake. For the db/db mice, the results were significant at p = 0.04 (n = 10 for both untreated and BCG treated groups). For the BALC/c control mice that also were treated in vivo with BCG under the same conditions, the results trended higher for the BCG-treated group, but nonsignificantly so (p = 0.31; n = 4 for both untreated and BCG treated groups).

## Discussion

Microbe-host interactions have systemic effects on host metabolism. Both mycobacteria *Mycobacterium tuberculosis* and *Mycobacterium bovis* as the BCG vaccine systemically influence sugar metabolism (Arts et al., 2016; Cheng et al., 2014; Gleeson et al., 2016; Kühtreiber and Faustman, 2019; Kühtreiber et al., 2018, 2020; Shi et al., 2015). Here we began by comparing the effects of repeat BCG vaccinations in a pilot clinical trial of adults with T1D and LADA. Using the Tokyo BCG strain, the two-year clinical trial demonstrated that subjects with T1D but not LADA had a statistically significant drop in HbA1c. This finding buttresses additional clinical trial data that now the Tokyo strain and Sanofi strain of BCG lowers HbA1c values in T1D (Kühtreiber et al., 2018). In monocyte culture, T1D had the best and largest increase in stimulated sugar transport after BCG exposure; LADA showed sluggish BCG-induced sugar transport, and this resulted in no clinical response. Despite the deficiency or compensation, sugar transport was stimulated by 24-hour BCG exposure using the glucose uptake assay. We broadened our investigation to ask whether other forms of diabetes, such as T2D, exhibited accelerated sugar uptake with this assay. We found monocytes from all forms of diabetes as well as NDCs responded to BCG in culture with accelerated sugar utilizations to varying degrees. Only in LADA was there an inherent minimal responsiveness. In mouse models, the T2D mouse model was dependent on more doses of BCG, but then responded similar to the T1D mouse model with lowered blood sugars. The universality of the effect implies that perhaps more forms of diabetes are likely to respond to BCG for therapeutic purposes by BCG augmented glycolysis, but the dosing and the time frame could vary. The data for both in vivo and in vitro BCG sugar metabolism effects in human and mice are summarized (Table 1).

**Table 1:** Diverse forms of diabetes: BCG responses *in vivo* and *in vitro*

BCG *in vivo*	Humans	Humans	Humans	Humans	Mice	Mice
	Diabetic	Diabetic	Diabetic	Controls	Diabetic	Diabetic
	T1D	Ldada	T2D	Control	T1D (NOD)	T2D (db/db)
Age	Adults	Adults	Adults	Neonates	Adolescents	Adolescents
Route	ID	ID	Bladder	ID	ID	ID
Strain	Tokyo/Pasteur	Tokyo	TICE	Diverse	Tokyo	Tokyo
HbA1c	↓	None	NA	NA	↓	↓
Stimulated sugar transport	Augmented	Reduced	NA	Augmented	Augmented	Augmented
**BCG *in vitro***	**T1D**	**LDADA**	**T2D**	**Control**	**T1D (NOD)**	**T2D (db/db)**
Stimulated sugar transport	Large response	Small response	Normal response	Normal response	NA	NA

The longevity of the BCG vaccine's systemic metabolic changes was evaluated by studying adults with decades-prior neonatal BCG vaccination. Remarkably, on average 55 years later, but extending up to 83 years, newborn BCG vaccinations appear to yield a lymphoid system with accelerated glucose utilization similar to those in more recently vaccinated human adults. The long-term durability of BCG effects on metabolism and parallel data sets documenting BCG effects on metabolism affecting DNA methylation patterns in glycolysis pathways are consistent with observed stability of the effects over decades (Kühtreiber et al., 2020).

A topic of broad interest is whether BCG strains can make a difference in clinical outcomes, especially outcomes beyond tuberculosis protection (Anderson et al., 2012; Davids et al., 2006; Ponte et al., 2018). For this pilot human clinical trial, the Tokyo BCG strain was utilized. Our earlier T1D clinical trials used the Sanofi BCG strain (Faustman et al., 2012; Kühtreiber et al., 2018). Both the Sanofi and Tokyo strains of BCG elicit comparable metabolic and strong immune responses (Abdallah et al., 2015; Angelidou et al., 2020). Equivalence in vivo of BCG strains has not always been observed in relation to tuberculosis protection as well as protection against off target infections (Shann, 2015). In bladder cancer comparison studies where high-dose BCG is used, the Sanofi strain of BCG was superior to the TICE strain (Rentsch et al., 2014). In our current study, high-dose TICE for bladder cancer had no systemic effect on glucose metabolism, even when the exposures were six weekly high doses of BCG. In the case of T1D, the TICE strain used in new-onset T1D in the past also failed to alter the diabetic disease course (Allen et al., 1999). In contrast, the Moreau and Sanofi/Pasteur strain of BCG seemed to slow or prevent diabetes disease (Karaci, 2014; Parent et al., 1997). A recent epidemiology study from BCG vaccination practices in Israel also supports the hypothesis that in human neonatal vaccinations with BCG, boosting doses has a protective impact on T1D, an effect observed with the Moreau strain (Klein, 2020). Data from Turkey, using the Russia BCG strain, show that children receiving three BCG vaccinations have statistically lower incidence of T1D than those receiving no or single BCG vaccination (Karaci, 2014). Tuberculosis itself protects from both T1D and the onset of multiple sclerosis but is hardly a clinically relevant pathway for therapy (Airaghi and Tedeschi, 2006; Andersen et al., 1981). Lastly, a recent study in the elderly showed no protection from COVID-19 infections with the Danish strain of BCG (Giamarellos-Bourboulis et al., 2020). These older Dutch citizens likely were also all vaccinated with BCG as newborns based on health practices, so the placebo group in this clinical trial likely had lingering protection, making it difficult to see a difference in the recently revaccinated BCG citizens to the previously vaccinated placebos. The data in this study show neonatal vaccinations as it relates to sugar transport continue to provide this high sugar uptake phenotype for decades as adults.

Will the BCG vaccine control blood sugars for T2D? The answer can only come from double-blinded clinical trials. Still, the data reported here provides cause for optimism. After BCG culture, T2D lymphoid cells at baseline were on average showing augmented sugar transport in a manner similar to monocytes from NDC subjects. In T2D, there is no underlying defect that blocks this metabolic effect of BCG and, unlike LADA subjects. The obese T2D mice did respond to BCG in vivo, but the dosing needed for this correction was much higher. Fewer BCG doses were needed in the T1D NOD mouse models. After in vivo treatment, the lymphoid cells harvested from the both obese db/db and autoimmune NOD treated mice had accelerated sugar transport. LADA human subjects who often have traits of T2D as well as T1D did not respond to BCG vaccinations in vivo over a two-year time course; longer-term follow-up studies are underway. BCG vaccination in children protects American Indian populations from T2D, according to a study using early versions of the Sanofi/Pasteur strain (Usher et al., 2019). Those American Indians were vaccinated as adolescents, before the start of metformin therapy and before a diagnosis of T2D. This may be an important aspect for achieving clinical trial success with BCG vaccinations in any form of diabetes; coadministration of metformin blocks the innate and adaptive immune training. We observe this again with BCG culture experiments and in the NOD experiments. Two mouse models show BCG can lower plasma cholesterol levels and delay atherosclerotic lesions in mice and eliminate the nonalcoholic fatty liver disease in obese diabetic ob/ob mice (Inafuku et al., 2015; Ovchinnikova et al., 2014; van Dam et al., 2016). Further follow-up will be needed in the clinic to achieve clear answers for T2D diabetes with attention to BCG doses, the time course of the responses, exclusion of metformin use at the time of dosing, time course for glycolysis activity, the responses, and the BCG strain.

In therapeutic interventional trials in T1D and also autoimmune diseases such as multiple sclerosis, the BCG effects, measured in terms of clinical outcome, all occur at about 2 years after the vaccinations. Recent evidence may help to explain the clinical observations that BCG as well as tuberculosis in humans (and mice) display such gradual clinical effects. Microbe BCG effects change both cytokines and glycolysis pathways. It is now appreciated that these host systemic effects are due to the microbe changing gene expression through methylation pattern rewiring. These processes take time and thus may explain the slow time course for the highly desirable and apparently permanent BCG effects on the immune system that may involve the bone marrow stem cells (Cirovic et al., 2020; Das et al., 2013; Kaufmann et al., 2018; Mitroulis et al., 2018). This BCG biologic journey may explain the delay in full clinical effects. These new discoveries may also help to explain early data in infection protection where the mechanisms of BCG actions were described as due to innate immunity, i.e., the use of monocytes and natural killer cells. If BCG does indeed end up reprogramming stem cells, then the BCG effects on glucose uptake can be observed in T cell lineages and also in monocyte lineages. Our data on BCG vaccines in humans show T cells are reprogrammed with BCG vaccinations, both in the setting of induction and modeling of the DNA by methylation changes and glucose metabolic changes. Adaptive training may be delayed, and the consequence of bone marrow retraining not just peripheral cytokine changes. Also, the 50+ year durability of prior BCG vaccinations conferring continuing metabolic changes suggests the slow onset of the metabolism if offset by decades long durability and perhaps an effect on stem cells.

Our pilot human clinical trial over a two-year time period does not address if LADA subjects will respond with slower kinetics. Certainly a longer time for clinical observations and trial durations will be needed. Monocytes from LADA monocytes in culture have very different metabolism of sugar compared with T1D monocytes. T1D monocytes with and without BCG have a very augmented sugar transport index; monocytes from LADA subjects are less responsive (Table 1). In many ways, these results are consistent with the hygiene hypothesis (Strachan, 1989). This hypothesis is rooted in the observation that subjects at most risk to allergies and autoimmunity have lessened microbe exposures. The fact that T1D is very responsive to this form of microbial exposure might suggest low past exposures to microbes that with exposures in vitro or in vivo augment glycolysis. The fact that LADA patients' onset of diabetes is delayed for 20+ years might mean they have had some past microbe exposure that corrected the underlying glycolysis defect, a delay in the disease onset. These data are consistent with the BCG vaccine prevention data where more BCG vaccines in childhood either outright prevent T1D or delay its onset (Karaci, 2014; Karaci and Aydin, 2012). Because all subjects in this study have never had BCG vaccinations, the possible microbe or other environmental factor providing this partial delay in disease onset is unknown.

### Limitations of the study

The limitations of this study include the need to directly test multiple BCG vaccinations in the setting of T2D with the correct BCG strain and to perform extended studies using LADA subjects to see if in vivo BCG responsiveness in glucose metabolism can occur. Like any new application of a therapy, the success lies in the details of the BCG strain, the correct BCG dosing, and also the selection of subjects most responsive. This study introduces the 2-NBDG assay as a monitoring tool for both human clinical trial data and also NOD mouse treatment data. The metformin blocking experiments serve to validate the utility of the assay and the need to carefully design clinical trials in T2D with an awareness of concurrent medications. The assay may detect the most responsive diabetic populations and early therapeutic effectiveness. Lastly, the ability of metformin to both block in vitro and in vivo BCG-induced glycolysis confirms the critical link of mammalian target of rapamycin (mTOR) regulated metabolism for BCG effectiveness in lowering blood sugars.

## STAR★Methods

### Key resources table

**Table:** 

REAGENT or RESOURCE	SOURCE	IDENTIFIER
**Antibodies**		

APC anti-Human CD14 antibody	BD Biosciences, Franklin Lakes, NJ	Cat# 555399

**Biological samples**		

Human primary monocytes (isolated form whole blood)	T1D, T2D and NDC volunteers	N/A

**Chemicals, peptides, and recombinant proteins**		

BCG	Japan BCG Laboratory, Tokyo, Japan	Lot# 1664-1

**Critical commercial assays**		

2-NBDG	ThermoFisher Scientific, Waltham, MA	Cat# N13195
BD FACSDiva CS&T Research Beads	BD Biosciences, Franklin Lakes, NJ	Cat#655051
CountBright Absolute Counting Beads	ThermoFisher Scientific, Waltham, MA	Cat# 2237596
Seahorse XF RPMI, pH7.4	Agilent Technologies, Santa Clara, CA	Cat# 00620006
FACScanto II	BD Biosciences, Franklin Lakes, NJ	Cat# 338960

**Experimental models: organisms/strains**		

NOD	The Jackson Laboratory, Bar Harbor, ME	Stock# 001976
db/db	The Jackson Laboratory, Bar Harbor, ME	Stock# 000697
Balb/c	Charles River Laboratories, Wilmington, MA	Strain# 028

**Other**		

BD Vacutainer K2 EDTA Plus Blood Correction Tubes	BD Biosciences, Franklin Lakes, NJ	Cat# 365900
Goldenrod Animal Lancet 5mm	Fisher Scientific, Waltham, MA	Cat# NC9416572
ACCU-CHEK Aviva Plus	Roche, Basel, Switzerland	Cat# 06988580001
ACCU-CHEK Aviva Plus 50 Test Strips	Roche, Basel, Switzerland	Cat# 06908349001
Mod LabDietR 5P00 with 0.1% Metformin	TestDiet, Richmond, IN	Cat# 1818831-214
BD 1mL Syringe	BD Biosciences, Franklin Lakes, NJ	Cat# 309628
PrecisionGlide Needle 30G1/2	BD Biosciences, Franklin Lakes, NJ	Cat# 305106
EasySep Direct Human Monocyte Isolation Kit	Stemcell Technologies, Cambridge, MA	Cat# 19669
Immunocult^TM^-SF Macrophage Medium	Stemcell Technologies, Cambridge, MA	Cat# 10961
Nunc UpCell 24 Multidish	ThermoFisher Scientific, Waltham, MA	Cat# NU24W1813
PBS, pH7.4	ThermoFisher Scientific, Waltham, MA	Cat# 20012-027
Easy 50 Magnet	ThermoFisher Scientific, Waltham, MA	Cat# 18001
EDTA	Sigma Aldrich, St. Louis, MO	Cat# E-7889
Penicillin-Streptomycin	ThermoFisher Scientific, Waltham, MA	Cat# 15140122
RBC Lysis buffer	ThermoFisher Scientific, Waltham, MA	Cat# 00-4333-57
RPMI	ThermoFisher Scientific, Waltham, MA	Cat# 61870036
Falcon™ Cell Strainers (40μm)	Corning, Glendale, AZ	Cat# 352340

### Resource availability

#### Lead contact

Further information and requests for resources and reagents should be directed to and will be fulfilled by the lead contact, Denise. L. Faustman (faustman@helix.mgh.harvard.edu).

#### Material availability

This study did not generate new unique reagents.

### Experimental model and subject details

#### Human experimental design

We undertook a single-center open-label pilot clinical trial conducted at the Massachusetts General Hospital (Boston, MA, USA). All human studies had full institutional approvals through Massachusetts General Hospital and Partners Health Care. The BCG interventional studies were also formally approved by the FDA (IND#2013P16434). The open-label patients were given two intradermal BCG vaccinations (Tokyo 172 BCG, Japan BCG Laboratory, Tokyo, Japan) at baseline and a second vaccine four weeks later. All blood donors, both T1D, T2D and non-diabetic control subjects, consented through Study #2001P001379. Informed consent was obtained from all subjects and the experiments conformed to the principles set out in the WMA Declaration of Helsinki and the Department of Health and Human Services Belmont Report. The timing of the serial blood sampling times for the pilot clinical trial was screening (pre-BCG vaccine), 6 months, year 01, year 1.5 and year 02.

All subjects, for inclusion into this pilot trial, had to be free at enrollment of significant diabetic complications such as retinopathy, amputations, cardiac disease, neuropathy as well as having no other co-morbidities, such as cancer, etc.

The pilot clinical trial reported here enrolled 16 diabetics; 6 of these diabetics (%Female of 0) had an early age of onset with a mean age of 11 ± 3 (duration of 18 ± 3 years; current age 28 ± 3). Ten of these diabetic subjects (%Female of 60) had a late age of onset with the mean age of onset of 31 ± 2 (duration of 19 ± 2 years; current age of 45 ± 4 years). A reference population of type 1 diabetic subjects, were also simultaneously followed (n = 40; %Female of 47.5) with an age of onset of 26 ± 2 (duration 13 ± 2 age of onset; current age of 40 ± 2 years). This data was compared to our earlier data on early age of onset subjects in the our Phase I clinical trial, dosed with the Sanofi strain of BCG, mean age of onset 11.3 ± 5.8 (current age 36.0 ± 2.1; duration of 24.7 ± 5.8 years) (Kühtreiber et al., 2018). For clarity throughout this paper we will refer to the two populations of diabetic subjects participating in the pilot as juvenile-onset T1D (AOO≤ 21 years) or adult-onset T1D subjects as Latent Autoimmune Diabetes in Adults (LADA) with AOO >21 years (Pozzilli and Pieralice, 2018). Percentage of female cohorts are 0 and 40.0 in juvenile-onset and LADA, respectively, therefore while no significant influence of sex is considered in LADA comparison (p = 0.7, Chi-square test), male-skewed cohorts could affect the results in juvenile onset T1D (p = 0.02).

The stimulated glucose index in T1D (n = 24), LADA (n = 12) and NDC (n = 16)-derived monocytes were also measured. Percentage of Female cohorts was 54.5, 31.6 and 50, respectively. Unfortunately, sex information of 3 T1D and one LADA cohorts were missing. Chi-square test were performed to see sex-related bias in these comparisons, finding that no significant difference at each comparison (NDC vs T1D, p = 0.3; NDC vs LADA, p = 0.7; T1D vs LADA, p = 0.2).

In this paper we also study type 2 diabetic (T2D) subjects with their typical traits of obesity, adult onset of diabetic disease and receipt of many forms of oral hypoglycemia agents as well as insulin therapy. This population had an average age of onset of 54.7 ± 3.0 years, disease duration of 15.2 ± 1.6 years, and current age of 70.1 ± 2.6; n = 13 subjects (%Female of 53.8). These were compared with NDC (n = 21; current age of 57.1 ± 4.2 years; %Female of 57.1) and/or T1D (n = 42, current age of 37.3 ± 2.8 years; age of onset of 20.0 ± 2.3 years; disease duration of 17.6 ± 2.1 years; %Female of 35.9) subjects in 2NBDG assay (Table S1). No significant difference in gender bias was found within individual comparisons (NDC vs T1D, p = 0.1; NDC vs T2D, p = 0.8; T1D vs T2D, p = 0.2, chi-square test), indicating no influence of sex on the assay. In addition, 13 of 21 NDC subject were neonatal BCG-vaccinated (%Female of 46.1), which also excluded sex-related bias in the comparison between them (p = 0.2).

We also studied 3 patients that received BCG treatment for bladder cancer (%Female of 0, p = 0.04). Thus, this result has a possibility of sex-linked influence. One of these patients was treated twice because of recurrent disease. The bladder cancer patients had an average BCG dosing age of 67.8 ± 2.6 years and an average age of 74.0 ± 2.4 years at the time of blood draw.

Blood from BCG-treated diabetic and non-diabetic control subjects (NDC) was collected in purple top tubes (BD Vacutainer, K_2_EDTA anti-coagulant) (Becton, Dickinson and Company; Franklin Lakes, NJ, USA). Sample HbA1c levels were processed directly from fresh blood by certified diagnostic laboratories approved by Massachusetts General Hospital.

#### Animals

Five-week-old BALB/c male, NOD female, and db/db male mice (The Jackson Laboratory, Bar Harbor, ME, USA) were housed under specific pathogen-free conditions. The mice were carefully monitored daily freely fed on normal diet and were not fasted before a challenge or assessment. When the mice showed signs of cachexia such as prominent loss of body weight or became moribund, they were immediately euthanized. The care of mice and experimental procedures were complied with the ‘‘Principles of Laboratory Animal Care’’ (Guide for the Care and Use of Laboratory Animals, National Institutes of Health publication 86-23, 1985). The experimental protocol was approved by the Institutional Animal Care and Use Committee of Massachusetts General Hospital (Approval protocol# 2017N000137).

The mice were housed in five animals per cage at the MGH animal facilities (four animals per cage after they reached a weight of >25 grams). Body weight and blood sugar measurements were taken weekly to monitor diabetes progression. Blood was obtained from tail nicks using a 5.5 mm animal lancet (MEDIpoint; Mineola, NY) and glucose was measured using an Aviva Plus glucometer (Accuchek; Indianapolis, Indiana). BCG was injected by footpad at a dose of 25 μL of BCG (2mg/ml in saline; Japan BCG Laboratory; Tokyo, Japan). Treated and untreated mice were sacrificed using CO_2_ and bone marrow was isolated from femurs and purified with RBC Lysis buffer (Thermofisher Scientific, Waltham, MA). One million cells were resuspended in 50μL Agilent Seahorse XFp media and mixed with 50 μL of 2-NBDG (0.2 mM in XFp, (Agilent, Santa Clara, CA, USA) . The suspension was incubated in a CO_2_-free incubator at 37°C for 30 minutes, washed and resuspended in 300 μL XFP. The cells were analyzed on a FacsCanto II flow cytometer. Data was analyzed using FlowJo software.

NOD mice (Jackson Labs; Bar Harbor, ME) were used as a model for T1D. A subgroup of NOD mice were started on metformin (0.1%) mixed into mouse chow (Scott Pharma Solutions; Marlborough, MA) at 6 weeks of age. After one month, half of the mice in the metformin group, and half of the mice in the control group were injected a single time with BCG. NOD mice were euthanized at 16–18 weeks of age and their bone marrow cells were harvested for 2NBDG assay. BALB/c and BKS db/db mice (Jackson Labs; Bar Harbor, ME) were used as models for non-diabetic control and T2D, respectively. Early experiments with the db/db mouse revealed a single injection of BCG at 7 weeks of age was insufficient to change blood sugar or weight, four BCG injections in alternating rear footpads were administered when the mice were 8, 14, 20 and 23 weeks old (data shown). Also, these mice were euthanized at 26–28 weeks of age.

### Method details

#### Monocyte isolation and culture

Whole blood samples were collected from human participants and monocytes were isolated using the StemCell Technologies Direct Human Monocyte Isolation kit (Stemcell Technologies, Vancouver, BC, Canada) and cultured overnight at 37°C and 5% CO2. The culture media was Immunocult media (Stemcell Technologies) the cells were placed into 24-well Nunc UpCell culture plates (Thermofisher Scientific, Waltham, MA) at 1 × 10^6^ monocytes per well. Culture conditions included with or without BCG (1 × 10^6^ CFU/well, BCG Japan, Tokyo, Japan) and with or without metformin (1 mM, Sigma Aldrich, St. Louis, MO). UpCell plates have temperature-responsive surfaces that allow cells to attach at 37°C, but release the cells at room temperature or below. To harvest the cells, the warm culture media was replaced with 1 mL of cold XFP buffer (Agilent Technologies, Santa Clara, CA) and the plates left at room temperature for 20 min to allow the cells to detach. The cells were then harvested and transferred to Eppendorf tubes.

#### 2-NBDG sugar transport assay

Harvested monocytes were washed with XFP buffer, counted, resuspended at 250K monocytes in 50 μL of XFP, mixed with 50 μL 2-NBDG in XFP (ThermoFisher, end concentration 0.1 mM) and incubated at 37°C for 20 min in a CO_2_-free incubator. Five μL of APC anti-human CD14 antibody (Company, City, State) was then added and incubated at 37°C for an additional 10 min. Unstained autofluorescence control samples were incubated without 2-NBDG and without CD14 antibody. Following incubation, the cells were washed with XFP buffer, resuspended in 300uL of XFP buffer and analyzed on a FacsCanto II flow cytometer (BD Biosciences, San Jose, CA). Data was analyzed using FlowJo software (FlowJo LLC, Ashland, OR; division of Becton, Dickinson & Company) by quantifying 2-NBDG fluorescence intensity (MFI).

Human primary monocytes were isolated using EasySep™ Direct Human Monocyte Isolation Kit (Stemcell Technologies, Cambridge, MA) following the manufacturer's protocol. Briefly, 1000 μL of Isolation Cocktail and 1000 μL of RapidSpheres were mixed with 20 mL of whole blood in a 50 mL centrifuge tube and incubated for 5 min at room temperature. Thirty milliliter of PBS containing 1mM EDTA (Sigma Aldrich, St. Louis, MO) was added and the tube placed into an “Easy 50” magnet. After 10 min, the monocytes-enriched suspension was transferred into a new tube, and then the magnetic separation process was repeated for 5 min with fresh, same amount RapidSpheres. The resulting highly monocytes-enriched suspension was transferred into another new tube and purified for a third time using the magnet.

The isolated 1 × 10^6^ human monocytes were cultured in 1mL Immunocult^TM^-SF Macrophage Medium (Stemcell Technologies) supplemented with 100 U/mL penicillin, 100 mg/mL streptomycin (ThermoFisher Scientific). Half of the wells were incubated with 1 × 10^5^ CFU of BCG (JAPAN BCG Laboratory, Tokyo, JAPAN) and the others with normal culture medium for control at 37°C in 5% CO_2_ and 95% air. The following day, the cells were harvested and analyzed by 2-NBDG assay.

### Quantification and statistical analysis

#### Data analysis

Statistical significance in the 2-NBDG experiments was determined by Student's t test to compare two groups. p values less than 0.05 were considered statistically significant. Pairing and number of tails used in the t tests are listed in the legend of the figures. Statistics were performed in Excel or GraphPad PRISM version 9. Levels of significance are shown in the figures using the following key: p < 0.05 ∗; p < 0.01 ∗∗; p < 0.001 ∗∗∗, p < 0.0001∗∗∗∗.

## Data Availability

All data reported in this paper and any additional information will be shared by the lead contact upon request. This paper does not report original code. Additional resource: The clinical registry number is NCT02081326 (https://clinicaltrials.gov/ct2/show/NCT02081326).
