# Supplementary material for: Bacillus Calmette-Guerin 's beneficial impact on glucose metabolism: evidence for broad based applications
Source: iScience. 2021 Sep 21;24(10):103150. doi: 10.1016/j.isci.2021.103150 (PMC8501688; doi:10.1016/j.isci.2021.103150)
Supplement: Document S1. Figures S1, S2 and Table S1 [file mmc1.pdf]

iScience, Volume *24*

## Supplemental information

# Bacillus Calmette-Guerin 's beneficial

# impact on glucose metabolism:

# evidence for broad based applications

**Gabriella F. Shpilsky, Hiroyuki Takahashi, Anna Aristarkhova, Michele Weil, Nathan Ng, Kacie J. Nelson, Amanda Lee, Hui Zheng, Willem M. Kühtreiber, and Denise L. Faustman**

Figure S1

## Bladder Cancer Patients treated with BCG

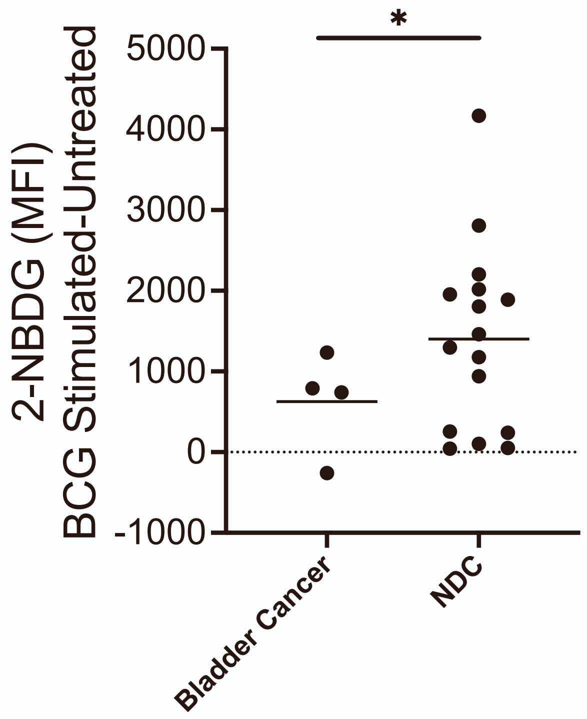


| Subject | Birth Country | BCG strain | Current Age | Dosing Age |
|---|---|---|---|---|
| NDC1 | US | TICE | 71 | 69 |
| NDC1 | US | TICE | 71 | 71 |
| NDC2 | US | TICE | 73 | 71 |
| T2D | US | TICE | 81 | 60 |

**Figure S1. Adult BCG bladder treatment does not augment peripheral monocyte glucose metabolism at least with the TICE BCG strain, Related to Figure 3.**
Glucose uptake in monocytes of NDC patients (n=16) following a one day incubation with or without BCG in culture compared to three elderly subject receiving high dose BCG for bladder cancer (n=3). One bladder treated subject was studied on two occasions; two of the subjects were NDC; and one bladder subject had T2D. The sugar uptake is presented as the difference from baseline to stimulated glucose uptake of monocytes in culture with BCG. All subjects were treated for bladder cancer with the TICE strain of BCG. Subjects' characteristics when the monocyte samples were studied at bottom of the figure. Statistics: Student's T test, one tailed, unpaired, with Welch's Correction $p < 0.05$ *

# Figure S2

T1D with juvenile onset
Age of Onset <= 21 years

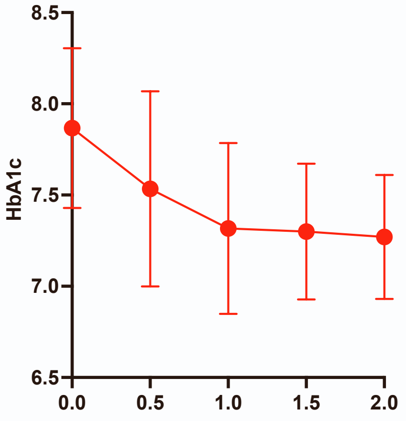


LADA
Age of Onset > 21 years

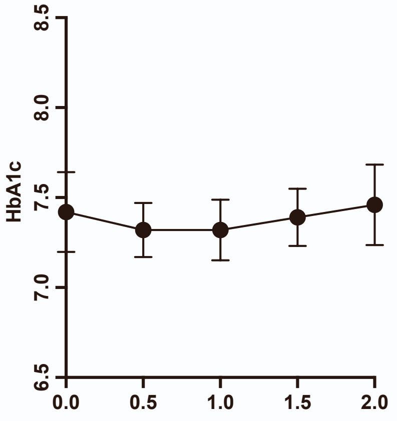


Reference

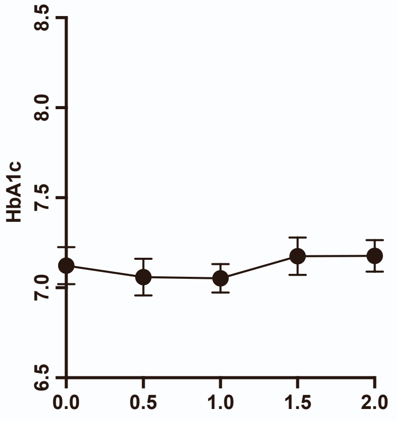


**Figure S2. Raw HbA1c data after in vivo BCG vaccinations in T1D and LADA patients, Related to Figure 1.**

This figure shows the decrease in HbA1c as averaged raw data and accompanies Fig 1A (which shows the same data as %change from baseline). Left: Juvenile-onset T1D diabetic subjects (n=6) treated with BCG as compared to an untreated reference T1D population (n=40). Right: LADA diabetic subjects (n=10) treated with BCG as compared to an untreated reference T1D population (n=40). See the legend for Figure 1 for further details.

# Table S1

| Cohort | Age | Age of Diabetes Onset | Duration | %Female |
|---|---|---|---|---|
| T1D | 37.3 ± 2.8 | 20.0 ± 2.3 | 17.6 ± 2.1 | 35.9 |
| T2D | 70.1 ± 2.6 | 54.7 ± 3.0 | 15.2 ± 1.6 | 53.8 |
| NDC | 57.1 ± 4.2 | N/A | N/A | 57.1 |

**Table S1. Demographics for 2-NBDG patients, related Figure 2-5.**
